# Sexual Function of Postmenopausal Women Addicted to Alcohol

**DOI:** 10.3390/ijerph15081639

**Published:** 2018-08-02

**Authors:** Anna Jenczura, Mariola Czajkowska, Agnieszka Skrzypulec-Frankel, Violetta Skrzypulec-Plinta, Agnieszka Drosdzol-Cop

**Affiliations:** 1 Chair of Woman’s Health in Katowice, School of Health Sciences in Katowice, Medical University of Silesia, 40-752 Katowice, Poland; annajenczura@interia.pl (A.J.); czajkowskamariola@op.pl (M.C.); skrzypulec-plinta@o2.pl (V.S.-P.); 2 Department of the Internal Disease, Dermatologia and Allergy, European Center of Diagnosis and Treatment of Urticaria, School of Medicine in Zabrze, Medical University of Silesia, 40-752 Katowice, Poland; agnes.skrzypulec@gmail.com

**Keywords:** alcoholism, menopause, sexual functioning

## Abstract

*Introduction*: Alcoholism impairs female sexual functions (decreased sex drive, reduced vaginal fluid, difficulty to experience orgasm). Aim: The aim of this study was to evaluate the course of perimenopausal period and sexual life of female alcoholics. *Methods*: 97 women at the age of 40–60 years were qualified with a diagnosed history of alcoholism (studied group). For the control group, 92 women at the age of 40–60 without a history of alcoholism were qualified. Questionnaires assessing female sexual function (Female Sexual Function Index—FSFI), the severity of perimenopausal symptoms (Menopause Rating Scale—MRS) and the degree of alcohol addiction (Michigan Alcoholism Screening Test—MAST) were obtained from each participant. *Main outcome measures*: To assess the sexual function of perimenopausal women addicted to alcohol. Results: The studied and control groups were significantly different (*p* = 0.00) in terms of severity of menopausal symptoms (MRS). The research showed lower quality of sexual life (FSFI) of women from the studied group. *Conclusions*: Population of women with diagnosed alcohol dependence enter the menopausal stage at the similar age as the population of female non-drinkers. The time of alcohol abuse is an important factor having an impact on both the course of menopause and the dynamics of the perimenopause period, leading to symptom escalation. The population of female alcoholics shows a decrease in sexual activity and the prevalence of sexual disorders.

## 1. Introduction

The State Agency for Prevention of Alcohol Related Problems estimates that the number of Polish people addicted to alcohol is about 800,000 (2% of the population). About 5–7% of the population (2–2.5 million people) drink alcohol in a way which is harmful to their health and poses a risk of developing addiction. Abuse of alcohol relates to 9% of the population and leads to about 10 thousand deaths per year. About 1/6 of all alcoholics enter rehabilitation [1].

Regardless of sex, alcoholism causes changes in the endocrine system. It has been proved that long-term alcoholism leads to ovarian atrophy by lowering the concentration of the growth hormone and luteinizing hormone (LH). Hormone changes in women cause masculinizing effects such as facial hair above the upper lip and a low voice. Alcohol abuse negatively affects the release of thyroid hormones, lowering the level of triiodothyronine, and adrenal cortex, where one can observe the increased level of cortisol. Long-term alcoholism leads to earlier onset of perimenopausal symptoms [2]. There are no detailed data relating to the age of female alcoholics at which the first menopause symptoms start or hormone economy in the postmenopausal period.

There are two types of mechanisms leading to sexual dysfunctions in people using psychoactive substances. Sexual problems may be a trigger for alcohol craving to reduce anxiety, psychological tension and to overcome embarrassment [3,4,5,6]. Alcoholic beverages are also used to overcome shyness and to feel more attractive. On the other hand, alcohol becomes a motivator to seek more intense sexual behavior [3,4,5,6]. Sexual problems may influence alcohol craving and increase the risk of relapse. Alcohol dependence and sexual disorders such as paraphilia can also occur at the same time [5,6].

Sexual disorders of female alcoholics have not been thoroughly investigated. Beckman’s study showed that alcohol abuse negatively affects the menstruation cycle and leads to changes manifested by increased sexual desire [7]. Excessive drinking often leads to risky sexual behaviors such as criminal offenses including sexual abuse [8].

It has been proved that sexual disorders in alcoholics are the effect of sexual exploitation from childhood and often incestuous relationships [9]. Long-term and excessive drinking leads to the deterioration of sexual performance. The most common sexual disorders are: decrease of sexual desire, the reduction of sexual response, sexual desire disorders, genital response disorders (lubrication and swelling of genitals and nipples), satisfaction and orgasm abnormalities [5,10].

## 2. Aims

The study results presented in the literature include generally the correlation of sexual life and alcohol use only among male populations. This observation became the basis for carrying out the present study, which aims to analyze the influence of alcoholism on the age of the last menstrual period occurrence, the dynamics and character of the perimenopausal period and sexual functioning among women.

## 3. Materials and Methods

### 3.1. Study Population

In total, 97 women with a diagnosis of alcohol addiction (by a physician) at the age of 40–60 years, remaining abstinent for at least one year, qualified for the study. They were sexually active, did not take anxiolytic agents and/or drugs reducing alcohol consumption, did not receive hormone treatment (oral contraceptives, Menopause Hormone Treatment—MHT), attended meetings organized by clubs, associations and self-help clinics for people addicted to alcohol and gave their informed consent and completed the questionnaire in full.

The exclusion criteria were the following: active drinking, no sexual activity, status post hysterectomy, the lack of informed consent or its withdrawal during the study, incomplete questionnaire.

Inclusion criteria for the control group were: women without recognized alcohol addiction (by a physician) aged 40–60 years, non-drinkers—abstinent or drinkers but who did not get 0 points in the MAST test, sexually active, not taking anxiolytic agents and/or drugs reducing alcohol consumption, not receiving hormone treatment (oral contraceptives, Menopause Hormone Treatment, MHT), not attending any meetings organized by clubs, associations and self-help clinics for people addicted to alcohol and who gave their informed consent and completed the questionnaire in full. For the control group we included 101 women aged 40–60 years who were living on the territory of Silesia in Poland. Finally, 92 women were qualified for the study as controls; 9 respondents were excluded from the project due to the high risk of relapse (MAST test analysis).

The research was carried out with the consent of the Bioethics Committee of the Medical University of Silesia in Katowice (No. of consent KNW/0022/KB/34/15).

### 3.2. Methods

The main tool was a self-designed questionnaire divided into three sections: sociometric, obstetrical and gynecologic, general (systemic diseases) and alcohol-related. It included 37 questions, which were closed, open and half-open.

MRS (Menopause Rating Scale), which is an extended version of a popular Kupperman Index and with added evaluation of psychosomatic symptoms. The scale includes 11 questions organized into 3 sub-groups of menopause symptoms: somato-vegetative, psychological and urogenital. The MRS has been standardized and adjusted (in many language versions, including Polish) to differentiate menopausal symptoms in women. The questionnaire possesses documented credibility, sensitivity, reliability, and internal consistency, as well as stability and repeatability of results in recognizing menopausal symptoms [11].

Somato-vegetative symptoms: hot flushes, sweating (episodes), cardiac complaints (irregular heartbeat, rapid heartbeat, chest tightness), sleeping disorders (difficulties in falling asleep, early wake up), joint and muscle discomfort (joint pain, rheumatic ailments).

Psychological symptoms: depressed mood (despondency, sadness, being close to tears, lack of motivation, mood swings), irritability (nervousness, inner anxiety, aggression), fear (inner anxiety, panic), physical and mental exhaustion lower general capabilities, deterioration of memory, poor concentration skills and forgetting) [11].

Urogenital symptoms: sexual problems (changes in sexual desire, activity and satisfaction), bladder problems (difficulty in passing urine, more frequent need to pass the urine, incontinence), vaginal dryness (the feeling of dryness or burning in the vagina, difficulties during sexual intercourse).

The points in each of the subcategories are given according to the scheme: no symptoms (0 point), mild (1 point), moderate (2 points), severe (3 points) and very severe (4 points). The composite score ranges from 0 (no menopause symptoms) to 44 (the highest extent of symptoms) and is the sum of all the above-mentioned symptoms of all sub-groups [11].

The Cronbach value of MRS for our results was estimated at the level of 0.911 for alcoholic women and 0.876 for controls, showing high reliability.

The Female Sexual Function Index (FSFI) developed by Rosen et al. [12] consists of 19 questions allowing for the multidimensional assessment of female sexual functions in relation to the period of the last 4 weeks. The index has been standardized and adjusted (in many language versions, including Polish) to differentiate sexual dysfunctions in women aged 18–70 in accordance with the current classifications and recommendations of scientific associations. The questionnaire possesses documented credibility, sensitivity, reliability, and internal consistency, as well as stability and repeatability of results in recognizing disorders of sexual desire, sexual arousal, orgasm, and dyspareunia.

The FSFI questionnaire is a multidimensional tool and contains 19 items organized in 6 domains (sub-scales). I—desire (2 questions), II—arousal (4 questions), III—lubrication (4 questions), IV—orgasm (3 questions), V—satisfaction (3 questions) and VI—pain (3 questions). The final results are achieved for each of the subscales by adding basic points from 6 domains and taking into account the assigned weight factor (0.6; 0.3; 0.3; 0.4; 0.4 and 0.4 for domains I–VI, respectively) to obtain the final score range of 0–6 points. The interpretation of the above partial scores is linear—the higher the score, the better sexual performance in each category.

One of the stages of this study was a global evaluation of the whole FSFI scale which provided a final score range of 2–36. A score of 26.55 points or below indicates clinically significant sexual dysfunctions in women [12].

The Cronbach value of FSFI for our results was estimated at the level of 0.985 for alcoholic women and 0.968 for controls, showing high reliability.

Michigan Alcoholism Screening Test (MAST), a diagnostic test for alcoholism consists of 24 questions, which evaluate the influence of alcohol intake on personal and family life and career and help to diagnose the symptoms and alcohol dependence criteria. The questions refer to the past 12 months. The questionnaire possesses documented credibility, sensitivity, reliability, and internal consistency, as well as stability and repeatability of results as a diagnostic test for alcoholism. A score of ≥5 indicates alcohol dependency. A score of 4 points is considered a dubious outcome. If the score is ≤3 the probability of alcohol dependency is very small [13].

The Cronbach value of MAST for our results was estimated at the level of 0.972 for alcoholic women and 0.931 for controls, showing high reliability.

### 3.3. Statistical Analysis

For statistical analysis, STATISTICA 10 PL (Statistica v.10, StatSoft Polska, Krakow, Poland) was used. A statistical significance level of *p* < 0.05 was used. CHI^2^ (or rather its modification in the form of Fisher’s test for contingency tables 2 × 2) was used to analyze the differences between the groups in regards to qualitative variables. To investigate the correlation between the above-mentioned parameters, Pearson’s linear correlation coefficient was calculated (when conformity with the normal distributions was confirmed).

## 4. Results

### 4.1. General Characteristics of the Study Groups

The average age of the respondents in the studied group was 52.43 ± 4.85 years, and in the control group: 51.29 ± 5.52 years. The difference was statistically insignificant. The average time of alcohol dependence in the studied group was 26.67 ± 8.93 years. In Table 1, the general characteristics of the studied and control groups are presented.

Studied and control groups were significantly different (*p* < 0.001) in terms of material status. In the studied group, one respondent in 10 reported poor material status. In the control group, more than half of respondents (53%) assessed their material status as good or very good in comparison to 30% in the studied group.

Significant differences were observed in regards to professional status. In the studied group, more than 95% of respondents worked in their acquired profession or another while in the control group the number was 75%.

The research showed significant differences for the two traits: civil status (*p* = 0.01300) and education (*p* = 0.01435). No statistical differences were observed for the places of residence among the two groups.

The age when the last period occurred was significantly different (*p* < 0.001) in the two groups. In the studied group, the last period was observed at the age of 49.18 ± 2.38 years while in the control group at the age of 50.92 ± 0.95 years. Moreover, statistical significance (*p* < 0.05) related to the age at which first menopause symptoms occurred. The age in the studied group was 45.02 ± 2.81 and in the control group 45.99 ± 1.77. The age of menarche was statistically insignificant between the groups.

### 4.2. Assessment of Menopausal Symptoms

The studied and control groups were significantly different in terms of severity of menopausal symptoms (MRS) in all aspects: psychological, somatic-vegetative, urogenital and in total. The respondents in the studied group reported menopause symptoms from moderate to very severe as opposed to the respondents in the control group (Table 2).

The analysis of the menopause symptoms gave conclusive results. Women in the studied group presented significantly higher mean values in all analyzed MRS symptoms (statistical significance *p* = 0.00) in comparison with the control group (Table 3).

### 4.3. Assessment of Sexual Function

The overall assessment of the FSFI scale and its 6 domains: sexual desire, arousal, lubrication, orgasm, satisfaction and pain showed that women in the studied group presented poorer sexual performance than women in the control group. Statistical significance of *p* < 0.05 was shown in each domain (Table 4).

A score of 26.55 points or below indicates clinically significant sexual dysfunctions in women. The detailed analysis of 6 domains of the FSFI questionnaire showed a statistical significance of *p* < 0.05 for each component.

A correlation between respondents’ age or time that has passed from their last period and the FSFI score was observed. It was negative in both cases: the older the respondent, the lower FSFI score; the longer the time from the last period, the lower the FSFI score. In the studied group the statistically significant correlation applied only to the level of respondents’ satisfaction (r = −0.2825, *p* = 0.008). The remaining correlations were negative but statistically insignificant. In the control group all FSFI domains (except for desire) significantly correlated with respondent’s age: the older the respondent, the lower FSFI score (Table 5).

## 5. Discussion

In recent years, one has observed a significant growth in the number of females drinking in a way that is harmful to their health. Their drinking habits are becoming more male-like. However, social stigma makes it difficult to carry research on female alcohol addiction [3,5,14,15]. Most the accessible research carried out around the globe is focused on male alcoholics only [15,16].

In bibliography related to the age of the last period in female alcoholics, authors only emphasize the fact that these women start menopause earlier [3,5]. No further information can be found in Polish or foreign literature. Research by Kaczmarek M. showed that the median age of menopause in Poland was 51.25, while the average age of the studied population was 49.5 [17]. In the studies carried out by Skrzypulec V. et al. among 1049 women at the age of 45–64 years (average age 51.31), the median age of menopause was 49.23 ± 4.37 [18]. Research by Barnaś E. et al. which covered 7183 females of various nationalities showed 49.2 years as the median age of the last period [19]. The results obtained by Gold et al. proved that the median age of menopause of American females was 52.54 years. The authors claim that the age of the last period is affected by: poor physical activity, lower level of education, hormonal contraception, smoking and alcohol intake [20]. This study showed that the last period in the studied group was at the age of 49.18 for the mean age of 52.43, and in the control group 50.92 for the age of 51.29. To sum it up, the results allow for the conclusion that menopause in the population of female alcoholics (remaining abstinent) does not occur significantly earlier than in non-drinkers. The results are contradictory to the previous literature claiming earlier menopause onset in female alcoholics. These reports seem to reflect common beliefs (no clinical studies) about alcohol’s destructive influence on the physiology of women’s body, and its aging in particular.

In 2013, Kryś-Noszczyk K. et al. carried out research on the severity of menopause symptoms among 210 Polish women at the age of 45–65 years. A number of menopause symptoms was presented, in the sequence from the most significant and characterized by the high level of severity. These included joint and muscle discomfort, depressive moods, sleeping difficulties, irritability, vaginal dryness and sexual problems. The calculated average of the results from all sub-groups (somatic-vegetative, psychological, urogenital) showed that psychological symptoms were most severe. Somatic-vegetative symptoms were the second followed by urogenital symptoms. Among all 11 MRS symptoms the most frequently reported as severe and moderate were hot flushes. Bladder problems and cardiac complaints were the least frequently reported [17]. This study proved its pioneering nature.

MRS results showed that females in both studied and control groups suffered most often from somatic-vegetative symptoms, then urogential followed by psychological menopause symptoms. Significantly higher values in three sub-groups in the studied group were observed, which show higher severity of some menopause symptoms. The same was observed when analyzing each of the 11 MRS questions separately [17]. The studies by Skrzypulec V. et al. related to the influence of menopausal symptoms on the functioning of perimenopausal women are confirmed by previous studies according to which hot flashes were listed as the most bothersome symptom [21]. At the same time, studies by Barnaś et al. show that according to the female population in Poland, most often reported complaints of the perimenopausal period are: nervousness, hot flashes, joint and muscle pains and insomnia [19].

Physiological changes that take place in women’s bodies with age significantly affect their sexual functions. Skrzypulec-Plinta et al. claim that approximately 40% of perimenopausal women report sexual problems [21]. The studies by Pauls R. et al. demonstrated that sexual dysfunctions are experienced by 43% of perimenopausal women [22]. The authors claim that the decrease of sexual activity is caused by the changes in the concentration of sex hormones such as estrogens, progesterone and androgens. The most frequent sexual disorders of the perimenopausal period are: libido decrease, sexual reluctance, the lack of arousal or pain during sexual intercourse [21].

When discussing endocrine status of alcoholic women, one needs to recount pioneering studies by Budzynski et al. In their clinical study, the concentration of sexual hormones in women with a history of alcohol dependence (during treatment) was measured throughout their menstruation cycles. In total, 50 women addicted to alcohol (mean age 39.8 ± 7.9 years) and 45 female non-addicts (42.8 ± 4.7 years) qualified for the study. The researchers investigated their biochemical liver parameters and measured their prolactin, foliculotropin, LH, estrogen and progesterone levels three times during their menstruation cycle: in the ovulation period, before and after their period. The results showed that female alcoholics had significantly lower LH and estrogen levels during ovulation. It has also been shown that liver dysfunction caused by alcohol abuse led to abnormal cyclic changes in the concentration of sexual hormones. Higher FSH and LH levels with similar estrogens, progesterone and testosterone levels in alcoholic females implied primary gonad failure and accelerated metabolism of steroids caused by microsomal enzyme activation [23]. The studies showed that liver dysfunction caused by previous alcohol abuse in the past might cause hormonal disorders in the perimenopausal period and later. The consequences may be manifested in many spheres, including endocrine and sexual.

Jarząbek G. et al. questions the common belief that estrogen production decreases sexual activity. The studies showed that a significant number of women had more intensive and satisfactory postmenopausal sexual life when they did not have to worry about menstruation and contraceptive limitations [24].

According to the research carried out in 2005 by Donnerstein, the age together with the low estrogen level negatively affect all sexual life domains, which leads to desire decrease and sexual dysfunction development [25]. A similar correlation was confirmed by studies carried out in 2000 in Massachusetts. In total, 200 peri- and postmenopausal women qualified for the study. It was proved that the interest in sexual activity diminishes with age, but the direct influence of estrogen level on all aspects of sex life was denied. It was also noted that reduced sexual activity of women in this period of life was often caused by their partner’s sexual dysfunctions [26]. Studies carried out by Blümel in 2004 also showed that women younger than 45 years stop their sexual activity because of erectile dysfunctions of their partners. Moreover, it was observed that women below 60 years of age gave up sexual activity because of their lowered sexual desire [27].

This study showed a correlation between women’s age (control and studied group) and their sexual functioning. The older the woman, the lower the score in all FSFI domains. Women below 45 years of age had the highest FSFI scores in all domains in comparison with women above 45 years of age. Scientific papers claim that poorer sexual function in this period is caused by the reduced blood flow in the vagina and vulva, which is the direct effect of blocking estrogen inflow, which increases sexual stimulation and vaginal lubrication [28,29]. Studies by Baldwin and Jenden proved that hormonal changes relating mainly to estrogen, FSH, testosterone and inhibin levels have a significant impact on vaginal lubrication and pain experienced during sexual intercourse [30]. The rise in sexual dysfunctions related to age was proved by the Penn Ovarian Aging Study carried out by Gracia et al. in 2007. Postmenopausal women experienced sexual disorders 2 or 3 three times more often than premenopausal women. It has also been noted that the age of the last period particularly affects experiencing an orgasm and vaginal dryness. The remaining causes of sexual dysfunctions included: the lack of a sexual partner, irritability, low DHES level or the presence of a child below 18 years of age [31]. According to Gonzălez, experiencing an orgasm by women is directly affected by: age, education level, emotional closeness with their partners and the level of vaginal lubrication [32].

This study showed a correlation between the time from the last period and FSFI scores. The longer the time from menopause, the lower the score in FSFI domains.

In 2008, Shifren observed that sexual disturbances are most common at the age between 45 and 64 and they are caused by a number of factors: health, low education level, depression, anxiety, thyroid diseases and incontinence [33]. The impact of health on sexual functioning has been also confirmed by studies carried out by Valadares A. et al. who researched 276 Brazilian women at the age of 40–60. The authors listed the most important factors disturbing women’s sexual lives: age, hot flashes, insomnia, depression nervousness, high blood pressure and incontinence [34].

This study’s overall assessment of the FSFI scale and its 6 domains revealed that women addicted to alcohol presented poorer sexual performance than non-alcoholic women. This was further confirmed by the detailed analysis of each FSFI question. One can assume that it is caused by excessive drinking and consequently, deterioration of general health conditions.

According to the studies by Gracia et al., greater fluctuation of testosterone concentration in women at the age of 35–47 lowers their desire in the perimenopausal period. Moreover, libido decrease is affected by clear fluctuations in the concentration of this hormone over time. Libido is also influenced by: depression and presence of kids in the place of residence [35].

According to western studies, statistical frequency of sexual intercourse is higher among women from southern Europe, which according to McCall and Meston is due to two factors positively affecting their sexual lives: emotional relationships with their partners and the change of sexual partners [36].

In recent years, one has observed a significant growth in the number of females drinking in a way that is harmful to their health. Their drinking habits are becoming more male-like, which can be cause by a common phenomenon of emancipation. Today’s focus on women’s health, which undoubtedly results from numerous feminist initiatives, points to the differences between sexes and the way of their interpretation when making diagnostic and therapeutic decisions. However, social stigma makes it difficult to carry out research on females’ alcohol addiction [3,5,14,15]. Most of the accessible research carried out around the globe is focused on male alcoholics only. This fact is confirmed by publications released by Trzebiatowski and Chodkiewicz [15,16].

In numerous scientific papers focusing on menopause, women’s sexuality and the quality of their life Polish and foreign authors tend to exclude from their research women with a history of alcohol dependence. Such disqualification can be also observed in the criteria of many other obstretrical-gyneological studies. This situation is reflected in popular clinical projects on hormonal therapies or menopausal hormonal therapies, in which history of alcoholism is usually an exclusion criteria (disqualification) from the study. Therefore, a low number of scientific papers including female alcoholics makes it extremely difficult to refer to the results of this study. To sum it up, there is a clear data gap which prevents the comparison of the overall results of this study with other similar outcomes.

We are aware of some limitations of this study. This is one of the first studies in Poland related to both the course of perimenopausal period and sexual life of female alcoholics—allowed for the specifying of risk factors for sexual disorders. First, the cross-sectional and not prospective nature of the study certainly does not allow for considering other factors which in the context of time could modify sexuality. Second, the study sample is too small to generalize the obtained results. Third, as in other self-reported inventories, the scoring systems are subjective in nature. Fourth, in the case of sexual disorders, the authors did not consider the lack of satisfaction as a factor discriminating the occurrence of clinically significant sexual disorders, although it seems that introducing such a scale would not affect the study results—the aim of the study was the assessment of the occurrence of disorders of particular sexual functions and not their intensity. Fifth, due to the inability to precisely determine the applied medications and their dosage, the connections between sexual functions and the applied pharmacological treatment have not been taken into consideration in the studied patients.

## 6. Conclusions

Women with a history of alcohol dependency (current abstainers) enter the menopausal stage at a similar age as the population of female non-drinkers. The time of alcohol abuse is an important factor affecting both the course of menopause and the dynamics of the perimenopausal period, leading to symptom escalation. Females with a history of alcohol dependence are characterized by a decline in sexual activity and sexual disorders in comparison with female non-drinkers.

## Figures and Tables

**Table 1:** General characteristics of the studied and control groups.

Trait		Studied Group *n* = 97	Control Group *n* = 92
Material status	Very good	5 (5.15%)	11 (11.96%)
	Good	24 (24.74%)	38 (41.3%)
	Average	59 (60.82%)	43 (46.74%)
	Poor	9 (9.28%)	0 (0.00%)
Professional status	Works in her acquired profession	23 (23.71%)	18 (19.57%)
	Works in profession other than acquired	50 (51.55%)	70 (76.09%)
	Receives a pension, does not work	7 (7.22%)	3 (3.26%)
	Receives a retirement pension, works	3 (3.09%)	0 (0.00%)
	Does not work	8 (8.25%)	1 (1.09%)
	Receives a disability pension, does not work	3 (3.09%)	0 (0.00%)
	Receives a disability pension, works	3 (3.09%)	0 (0.00%)
Place of residence	Rural area	1 (1.03%)	1 (1.09%)
	Urban area <20,000	0 (0.00%)	0 (0.00%)
	Urban area 20,000–100,000	54 (55.67%)	57 (61.96%)
	Urban area >100,000	42 (43.30%)	34 (36.96%)
Marital status	Married	61 (62.89%)	69 (75%)
	Divorced in a free non-marital relationship	14 (14.43%)	18 (19.57%)
	Widow in a free non-marital relationship	10 (10.31%)	2 (2.17%)
	Single in a free non-marital relationship	4 (4.12%)	1 (1.09%)
	Married with another partner	8 (8.25%)	1 (1.09%)
	Divorced, no partner	0 (0.00%)	1 (1.09%)
Education	Primary	1 (1.03%)	0 (0.00%)
	Vocational	28 (28.87%)	16 (17.39%)
	Secondary	40 (41.24%)	29 (31.52%)
	Bachelor/Engineer degree	10 (10.31%)	21 (22.83%)
	Master degree	18 (18.56%)	22 (23.91%)

**Table 2:** The characteristics of menopause symptoms (MRS scale) in the studied and control groups.

Symptoms (MRS Scale)	Studied Group *n* = 97 Mean ± SD	Control Group *n* = 92 Mean ± SD	*p*
psychological	7.08 ± 4.08	4.10 ± 2.81	0.000000
somatic-vegetative	9.20 ± 3.21	5.27 ± 2.66	0.000000
urogenital	8.04 ± 2.50	4.32 ± 2.31	0.000000
total	24.32 ± 7.85	13.68 ± 7.10	0.000000

**Table 3:** The evaluation of the severity of menopause symptoms (MRS scale) in the studied and control groups. (*—statistically significant).

Detailed Symptoms (MRS Scale)	Studied Group *n* = 97	Control Group *n* = 92	*p*
hot flushes, sweating (episodes)	2.62 ± 1.15 *	1.67 ± 0.93 *	0.000000
	Me = 3.00	Me = 2.00	
	Pe5: 0.00	Pe5: 0.00	
	Pe95: 4.00	Pe95: 3.00	
cardiac complaints (irregular heart beat, rapid heartbeat, chest tightness)	2.36 ± 1.10 *	1.09 ± 0.74 *	0.000000
	Me = 3.00	Me = 1.00	
	Pe5: 0.00	Pe5: 0.00	
	Pe95: 4.00	Pe95: 2.00	
sleeping disorders (difficulties in falling asleep, early wake up)	2.49 ± 1.05 *	1.22 ± 0.84 *	0.000000
	Me = 3.00	Me = 1.00	
	Pe5: 0.00	Pe5: 0.00	
	Pe95: 4.00	Pe95: 2.00	
depressed mood (despondency, sadness, being close to tears, lack of motivation, mood swings)	2.04 ± 1.08 *	1.03 ± 0.82 *	0.000000
	Me = 2.00	Me = 1.00	
	Pe5: 0.00	Pe5: 0.00	
	Pe95: 4.00	Pe95: 2.00	
irritability (nervousness, inner anxiety, aggression)	1.70 ± 1.18 *	1.07 ± 0.75 *	0.000020
	Me = 2.00	Me = 1.00	
	Pe5: 0.00	Pe5: 0.00	
	Pe95: 4.00	Pe95: 2.00	
fear (inner anxiety, panic)	1.55 ± 1.22 *	0.88 ± 0.83 *	0.000247
	Me = 2.00	Me = 1.00	
	Pe5: 0.00	Pe5: 0.00	
	Pe95: 4.00	Pe95: 2.00	
physical and mental exhaustion (generally lower capabilities, deterioration of memory, poor concentration skills and tendency to forget things)	1.79 ± 1.18 *	1.02 ± 0.77 *	0.000000
	Me = 2.00	Me = 1.00	
	Pe5: 0.00	Pe5: 0.00	
	Pe95: 4.00	Pe95: 2.00	
sexual problems (changes in sexual desire, activity and satisfaction)	2.54 ± 0.96 *	1.30 ± 0.90 *	0.000000
	Me = 3.00	Me = 1.00	
	Pe5: 1.00	Pe5: 0.00	
	Pe95: 4.00	Pe95: 3.00	
bladder problems (difficulty in passing urine, more frequent need to pass the urine, incontinence)	2.33 ± 1.12 *	1.42 ± 1.02 *	0.000000
	Me = 3.00	Me = 1.00	
	Pe5: 0.00	Pe5: 0.00	
	Pe95: 4.00	Pe95: 3.00	
vaginal dryness (the feeling of dryness or burning in the vagina, difficulties during sexual intercourse)	3.18 ± 1.13 *	1.59 ± 0.89 *	0.000000
	Me = 4.00	Me = 2.00	
	Pe5: 1.00	Pe5: 0.00	
	Pe95: 4.00	Pe95: 3.00	
joints and muscles discomfort (joint pain, rheumatic ailments)	1.72 ± 1.25 *	1.29 ± 0.83 *	0.006363
	Me = 2.00	Me = 1.00	
	Pe5: 0.00	Pe5: 0.00	
	Pe95: 4.00	Pe95: 2.00	

**Table 4:** The evaluation of sexual function in the studied and control groups—FSFI scale. (*—statistically significant).

Domains	Studied Group *n* = 97	Control Group *n* = 92	Total	*p*
Sexual desire				0.000000
Mean ± SD	5.78 ± 1.54 *	7.07 ± 1.20 *	6.41 ± 1.52	
Min–Max	2.00–10.00	3.00–10.00	2.00–10.00	
95% CI	5.47–6.09	6.82–7.31	6.19–6.63	
Arousal				0.000014
Mean ± SD	12.44 ± 3.49 *	14.26 ± 1.80 *	13.33 ± 2.93	
Min–Max	4.00–20.00	8.00–19.00	4.00–20.00	
95% CI	11.74–13.15	13.89–14.63	12.91–13.75	
Lubrication				0.000000
Mean ± SD	12.61 ± 3.61 *	15.07 ± 2.54 *	13.80 ± 3.36	
Min–Max	4.00–20.00	9.00–20.00	4.00–20.00	
95% CI	11.88–13.34	14.54–15.59	13.32–14.29	
Orgasm				0.000000
Mean ± SD	8.40 ± 2.53 *	11.04 ± 1.81 *	9.68 ± 2.57	
Min–Max	3.00–15.00	7.00–15.00	3.00–15.00	
95% CI	7.89–8.91	10.67–11.42	9.31–10.05	
Satisfaction				0.000045
Mean ± SD	9.46 ± 2.81 *	10.88 ± 1.69 *	10.15 ± 2.43	
Min–Max	3.00–15.00	7.00–15.00	3.00–15.00	
95% CI	8.90–10.03	10.53–11.23	9.80–10.50	
Pain				0.000000
Mean ± SD	7.96 ± 2.08 *	10.90 ± 2.15 *	9.39 ± 2.57	
Min–Max	3.00–13.00	7.00–15.00	3.00–15.00	
95% CI	7.54–8.38	10.46–11.35	9.02–9.76	

**Table 5:** The correlation of FSFI scores with respondents’ age and age of menopause in the studied and control group.

Domains:	Studied Group *n* = 97		Control Group *n* = 92	
	Age (years)	Age of Menopause	Age (years)	Age of Menopause
Sexual desire	−0.1537	−0.0848	−0.079	0.0575
	*p* = 0.153	*p* = 0.432	*p* = 0.495	*p* = 0.619
Arousal	−0.1996	−0.085	−0.2679	0.0717
	*p* = 0.062	*p* = 0.431	*p* = 0.018	*p* = 0.535
Lubrication	−0.2072	−0.1319	−0.3972	−0.0895
	*p* = 0.053	*p* = 0.221	*p* = 0.000	*p* = 0.439
Orgasm	−0.133	−0.0303	−0.2792	−0.0569
	*p* = 0.217	*p* = 0.780	*p* = 0.014	*p* = 0.623
Satisfaction	−0.2825	−0.1418	−0.2847	−0.0125
	*p* = 0.008	*p* = 0.188	*p* = 0.012	*p* = 0.914
Pain	−0.117	−0.165	−0.2999	−0.1708
	*p* = 0.278	*p* = 0.125	*p* = 0.008	*p* = 0.138
